# Angiotensin II treatment is associated with improved oxygenation in ARDS patients with refractory vasodilatory shock

**DOI:** 10.1186/s13613-023-01227-5

**Published:** 2023-12-16

**Authors:** Daniel E. Leisman, Damian R. Handisides, Lakhmir S. Chawla, Timothy E. Albertson, Laurence W. Busse, David W. Boldt, Adam M. Deane, Michelle N. Gong, Kealy R. Ham, Ashish K. Khanna, Marlies Ostermann, Michael T. McCurdy, B. Taylor Thompson, James S. Tumlin, Christopher D. Adams, Tony N. Hodges, Rinaldo Bellomo

**Affiliations:** 1 Department of Medicine, Massachusetts General Hospital, 55 Fruit St., GRB 7-730, Boston, MA 02114 USA; 2 Department of Anesthesia, Critical Care, and Pain Medicine, Massachusetts General Hospital, Boston, MA USA; 3 Innoviva Specialty Therapeutics, Waltham, MA USA; 4 Department of Medicine, Veterans Affairs Medical Center, San Diego, CA USA; 5 Departments of Medicine, Emergency Medicine and Anesthesiology, School of Medicine, UC Davis, Sacramento, CA USA; 6 Department of Medicine, Emory University, Atlanta, GA USA; 7 Emory Critical Care Center, Emory Healthcare, Atlanta, GA USA; 8 Division of Critical Care, Department of Anesthesiology and Perioperative Medicine, University of California, Los Angeles, Los Angeles, CA USA; 9 Department of Medicine and Radiology, Royal Melbourne Hospital, The University of Melbourne, Melbourne Medical School, Parkville, Australia; 10 Division of Critical Care Medicine, Division of Pulmonary Medicine, Montefiore Medical Center, Albert Einstein College of Medicine, Bronx, NY USA; 11 Department of Critical Care, Mayo Clinic, Phoenix, AZ USA; 12 Department of Anesthesiology, Section On Critical Care Medicine, Wake Forest University School of Medicine, Atrium Health Wake Forest Baptist Medical Center, Winston-Salem, NC USA; 13 Perioperative Outcomes and Informatics Collaborative (POIC), Winston-Salem, NC USA; 14 Outcomes Research Consortium, Cleveland, OH USA; 15 Department of Critical Care, King’s College London, Guy’s & St Thomas’ Hospital, London, UK; 16 Division of Pulmonary and Critical Care Medicine, Department of Medicine, University of Maryland School of Medicine, Baltimore, MD USA; 17 Department of Emergency Medicine, University of Maryland School of Medicine, Baltimore, MD USA; 18 Department of Medicine, Harvard Medical School, Boston, MA USA; 19 Renal Division, Department of Medicine, Emory University Medical Center, Emory University, Atlanta, GA USA; 20 Australian and New Zealand Intensive Care Research Centre (ANZIC-RC), School of Public Health and Preventive Medicine, Monash University, Melbourne, Australia; 21 Department of Critical Care, Melbourne Medical School, University of Melbourne, Austin Hospital, Melbourne, Australia; 22 Data Analytics Research and Evaluation (DARE) Centre, Austin Hospital, Melbourne, Australia; 23 Department of Intensive Care Medicine, Austin Hospital, Melbourne, Australia; 24 The Australian and New Zealand Intensive Care Society (ANZICS) Centre for Outcome and Resource Evaluation (CORE), Melbourne, Australia; 25 Intensive Care Unit, Royal Melbourne Hospital, Melbourne, VIC Australia

**Keywords:** Angiotensin II, Renin–angiotensin system, ARDS, Norepinephrine, Shock, Septic

## Abstract

**Background:**

The physiological effects of renin-angiotensin system modulation in acute respiratory distress syndrome (ARDS) remain controversial and have not been investigated in randomized trials. We sought to determine whether angiotensin-II treatment is associated with improved oxygenation in shock-associated ARDS.

**Methods:**

Post-hoc subgroup analysis of the Angiotensin Therapy for High Output Shock (ATHOS-3) trial. We studied patients who met modified Berlin ARDS criteria at enrollment. The primary outcome was PaO_2_/FiO_2_-ratio (P:F) at 48-h adjusted for baseline P:F. Secondary outcomes included oxygenation index, ventilatory ratio, PEEP, minute-ventilation, hemodynamic measures, patients alive and ventilator-free by day-7, and mortality.

**Results:**

Of 81 ARDS patients, 34 (42%) and 47 (58%) were randomized to angiotensin-II or placebo, respectively. In angiotensin-II patients, mean P:F increased from 155 mmHg (SD: 69) at baseline to 265 mmHg (SD: 160) at hour-48 compared with no change with placebo (148 mmHg (SD: 63) at baseline versus 164 mmHg (SD: 74) at hour-48)(baseline-adjusted difference: + 98.4 mmHg [95%CI 35.2–161.5], *p* = 0.0028). Similarly, oxygenation index decreased by − 6.0 cmH_2_O/mmHg at hour-48 with angiotensin-II versus − 0.4 cmH_2_O/mmHg with placebo (baseline-adjusted difference: -4.8 cmH_2_O/mmHg, [95%CI − 8.6 to − 1.1], *p* = 0.0273). There was no difference in PEEP, minute ventilation, or ventilatory ratio. Twenty-two (64.7%) angiotensin-II patients had sustained hemodynamic response to treatment at hour-3 versus 17 (36.2%) placebo patients (absolute risk-difference: 28.5% [95%CI 6.5–47.0%], *p* = 0.0120). At day-7, 7/34 (20.6%) angiotensin-II patients were alive and ventilator-free versus 5/47(10.6%) placebo patients. Day-28 mortality was 55.9% in the angiotensin-II group versus 68.1% in the placebo group.

**Conclusions:**

In post-hoc analysis of the ATHOS-3 trial, angiotensin-II was associated with improved oxygenation versus placebo among patients with ARDS and catecholamine-refractory vasodilatory shock. These findings provide a physiologic rationale for trials of angiotensin-II as treatment for ARDS with vasodilatory shock.

*Trial Registration*: ClinicalTrials.Gov Identifier: NCT02338843 (Registered January 14th 2015).

**Supplementary Information:**

The online version contains supplementary material available at 10.1186/s13613-023-01227-5.

## Introduction

Critically ill patients with acute respiratory distress syndrome (ARDS) frequently have associated vasodilatory shock requiring infusions of vasopressors. Angiotensin-II is a non-catecholamine endogenous hormone of the renin-angiotensin system (RAS) that elicits vasoconstriction. Synthetic angiotensin-II was approved to increase blood pressure in vasodilatory shock after the Angiotensin-II for the Treatment of High-Output Shock-3 (ATHOS-3) trial demonstrated the peptide’s efficacy as a vasopressor in catecholamine-refractory vasodilatory shock [1]. However, the biological and clinical effects of RAS modulation in patients with ARDS are controversial [2].

Inflammatory excess likely contributes to the progression of a meaningful subset of acute lung injuries that produce ARDS [3, 4]. While the angiotensin-II/type-1 receptor (AT1R) signaling axis has well established pro-inflammatory effects in chronic cardiorenovascular disease that could theoretically worsen ARDS, a randomized trial in critically-ill COVID-19 patients ended enrollment early due to high probability that RAS-inhibition caused harm [5]. On the other hand, experimental studies report divergent effects of catecholamines and angiotensin-II on the pulmonary vasculature [6, 7], and in COVID-19 ARDS, several observational studies independently reported increased systemic arterial oxygenation after the initiation of angiotensin-II treatment [8–11]. However, there are no randomized clinical trials of angiotensin-II in ARDS. Thus, the impact of angiotensin-II therapy in ARDS remains uncertain. Yet, determining if and how angiotensin-II impacts pulmonary function in ARDS would have immediate implications for both clinical practice and our understanding of disease biology.

Accordingly, to investigate whether angiotensin-II treatment is associated with clinically relevant effects on pulmonary function, we conducted a *post-hoc* analysis of ATHOS-3, a phase-III multicenter placebo-controlled, randomized clinical trial of angiotensin-II treatment in catecholamine-refractory vasodilatory shock [1]. We hypothesized that, compared to placebo, among patients with ARDS and refractory shock, angiotensin-II therapy would be associated with improved systemic oxygenation.

## Methods

### ATHOS-3 trial

The ATHOS-3 trial has been previously described (clinicaltrials.gov identifier NCT 02338843) [1]. Briefly, adults with persistent vasodilatory shock after ≥ 25 mL/kg of volume resuscitation requiring high-dose vasopressors (i.e., norepinephrine-equivalent dose (NED) > 0.2 μg/kg/min) were randomly assigned 1:1 to receive synthetic human angiotensin-II (La Jolla Pharmaceutical Co.) or saline placebo plus standard vasopressors. Randomization was stratified by mean arterial pressure (MAP) at screening and Acute Physiology and Chronic Health Evaluation II (APACHE-II) score. The trial was completed before the COVID-19 pandemic began.

### Objectives

The present study reflects a post-hoc analysis of ATHOS-3 in the subset of patients who had ARDS at enrollment that aimed to answer the following questions:

1. Was angiotensin-II associated with improved oxygenation independent of other differences in ventilatory support in ARDS?
2. What were the clinical outcomes of ARDS patients treated with angiotensin-II versus placebo?

### Patients

The present study included all ATHOS-3 participants who met modified Berlin criteria for ARDS at enrollment [12]. All patients had bilateral opacities on chest imaging, a PaO_2_/FiO_2_ ratio (P:F) ≤ 300 mmHg despite mechanical ventilation with ≥ 5 cmH_2_O of positive end-expiratory pressure (PEEP). To ensure the PEEP criterion was met, we included only patients receiving positive-pressure ventilation at enrollment. We refer to these criteria as modified because a volume overload component of hypoxemia could not be definitively excluded for all cases. However, low output states, defined as cardiac index < 2.3 L/min m^2^ or central venous oxygenation saturation (ScvO_2_) < 70% with central venous pressure (CVP) < 8 mmHg were exclusion criteria in ATHOS-3.

### Interventions

Study drug infusion was started at 20 ng/kg/min and titrated during the first 3 h to achieve MAP ≥ 75 mmHg while keeping other vasopressor doses constant. Thereafter, study drug and other vasopressors were titrated at treating clinicians’ discretion to maintain MAP between 65 and 75 mmHg. At 48 h, study drug infusion was discontinued according to a protocol-specified tapering process but could be continued for up to 7 days per clinician discretion.

### Respiratory physiologic outcomes

The primary aim of this study was to determine whether angiotensin-II treatment was associated with improved oxygenation versus placebo. The primary outcome for this analysis was P:F at 48 h post-treatment. As an additional measure of oxygenation, we determined the oxygenation index (OI), defined:

$${\text{OI}} = \frac{{{\text{FiO}}_{2} \times {\text{mAwP}}}}{{{\text{PaO}}_{2} }},$$

where mAwP = mean airway pressure. The OI is a validated measure of oxygenation in ARDS that, unlike P:F, also incorporates the level of ventilatory support [13, 14]. Lower OI corresponds to greater oxygenation.

As another measure of pulmonary function, we calculated the ventilatory ratio (VR), which reflects the degree of dead space ventilation [15], defined:

$${\text{VR}} = \frac{{{\text{PaCO}}_{2} \times {\text{minute}}\;{\text{ventilation}}}}{{{\text{Ideal}}\;{\text{body}}\;{\text{weight}}\; \times 100 \times 37.5}}.$$

Because improvements in oxygenation could be driven by changes in ventilator management independent of an effect of angiotensin-II, we also assessed PEEP, respiratory rate, tidal volumes, minute ventilation, PaCO_2_, and mAwP.

All pulmonary and ventilator measures were assessed at baseline, hour-3, and hour-48.

### Cardiovascular outcomes

The primary efficacy outcome of the ATHOS-3 trial was treatment response at hour-3, defined as a sustained increase in MAP, either > 75 mmHg or > 10 mmHg above baseline MAP, without an increase in background vasopressors [1]. We additionally assessed the total NED and the MAP over the 48-h study period.

### Exploratory vasopressor dose analysis

Experimental studies suggest angiotensin-II augments hypoxic pulmonary vasoconstriction (HPV) [16, 17]. Literature also suggests that catecholamines inhibit HPV [18, 19]. Therefore, an association of angiotensin-II treatment with improved oxygenation could either suggest a direct effect of angiotensin-II (e.g., by augmenting HPV) or an indirect effect of catecholamine dose reduction (e.g., by reducing inhibition of HPV). We explored these hypotheses, summarized in Additional file 1: Fig. S1, as follows.

Under the hypothesis that an association of angiotensin-II treatment with increased oxygenation predominantly reflects a catecholamine-sparing (i.e., indirect) effect, we hypothesized that:

- Adjusting for NED will attenuate the association of angiotensin-II with P:F.
- NED will be strongly associated with P:F over time.

Conversely, under the hypothesis that an association of angiotensin-II with increased oxygenation predominantly reflects a direct effect of treatment, we instead hypothesized that:

- Angiotensin-II treatment should remain associated with P:F after adjustment for NED
- NED should not be associated with P:F.

As sensitivity analysis, we performed this analysis first using the total vasopressor dose in norepinephrine-equivalents (NED_Total_), and again using only the catecholamine vasopressor dose (NED_Catechol_).

### Exploratory clinical outcomes

We anticipated the study to be underpowered to detect differences in clinical outcomes. However, as an exploratory analysis, we tabulated clinical outcomes between groups. Outcomes of interest included by day-7, whether patients were alive and liberated from the ventilator or alive and off vasopressors, respectively, and by day-28, whether patients had died or were alive and discharged from the hospital.

### Statistical analysis

Continuous variables are reported as mean (SD) or median (interquartile range) as appropriate, categorical variables as frequency (percent). Missing data management is detailed in Additional file 1: Supplemental Methods. Analyses were performed in SAS (SAS Institute, Cary, NC, USA.)

In the primary analysis, respiratory indices at hour-3 and hour-48 were modeled in a linear regression that included terms for treatment and baseline value of the dependent variable in accordance with best statistical practice for evaluating continuous outcomes in clinical trials [20, 21]. We also report mean differences between hour-48 and baseline measurements by treatment group, with 95%CI and *p*-values calculated using paired *T*-tests.

For multivariable analyses, we additionally included prespecified covariates that could potentially confound the outcome. The prespecified covariates were age, sex, BMI, and baseline APACHE-II score, MAP, NED, PEEP, and minute ventilation.

For the exploratory analysis of treatment and NED effects over time, we used longitudinal mixed-effects repeated-measures models. This approach was chosen to assess time-varying associations of NED with oxygenation and to account for within-subject correlation and the sequence of measurements. Individual patients were entered as random-effects and time was treated as a 3-level categorical repeated-measure variable with a compound symmetry covariance structure. The model included fixed-effects for treatment, NED as a continuous variable, and interaction terms for these effects with time. We iterated the model twice, using NED_Total_, and NED_Catechol_, respectively. To facilitate intuitive interpretation, least-squares means and errors were constructed and graphed at different levels of NED across timepoints and treatment groups.

For the exploratory clinical outcomes, we report the absolute risk-difference, relative risk, and 95% confidence intervals.

## Results

Of the 321 patients enrolled in ATHOS-3, 81 (25.2%) met Berlin criteria for ARDS at enrollment and were included in this study. Among these, 34 (42%) were randomized to angiotensin-II and 47 (58%) to placebo. The baseline characteristics are shown in Table 1. All patients were invasively ventilated at baseline. Sepsis was the likely or definite cause of shock in more than 90% of the cohort. Overall, the angiotensin-II group had more women (64.7%) than the placebo group (34.0%). The groups were otherwise well balanced for age, body-mass index, cause of vasodilatory shock, as well as baseline vasopressor support level, cardiac index, CVP, ScvO_2_, APACHE-II score, and albumin level.

**Table 1:** Baseline and treatment characteristics

Outcome	Placebo	Angiotensin-II	All Patients
*N*	47	*34*	*81*
Demographics and clinical factors			
Age (years)	60 (17)	57 (18)	59 (17)
Female—*n* (%)	16 (34%)	22 (65%)	38 (47%)
Body Mass Index (kg/m^2^)	30.2 (8.4)	28.9 (7.5)	29.6 (8.0)
Ideal body weight (kg)	63.6 (10.6)	59.1 (10.2)	61.7 (10.6)
Exposure to ACE inhibitors or ARBs—*n* (%)	5 (11%)	6 (18%)	11 (14%)
Cause of vasodilatory shock—*n* (%)			
Sepsis	41 (87%)	28 (82%)	69 (85)
Other—potentially sepsis	4 (9%)	4 (12%)	8 (10%)
Other—not sepsis	2 (4%)	2 (6%)	4 (5%)
Baseline APACHE II Score	30.9 (7.9)	29.1 (8.2)	30.1 (8.0)
Baseline albumin (g/dl)	2.4 (0.6)	2.3 (0.8)	2.3 (0.7)
Baseline cardiovascular status			
Mean arterial pressure (mmHg)	65 (7)	66 (5)	65 (6)
Average NED in past 6 h (µg/kg/min)	0.55 (0.32)	0.54 (0.35)	0.54 (0.33)
Vasopressin use in past 6 h—n (%)	40 (85%)	24 (71%)	64 (79%)
Central venous pressure (mmHg)	13.6 (4.5)	15.1 (5.3)	14.3 (4.9)
Cardiac Index (L/min/m^2^)	3.4 (0.9)	3.4 (0.7)	3.4 (0.8)
ScvO_2_ (%)	78 (7)	77 (8)	78 (8)
Baseline respiratory status			
PaO_2_:FiO_2_ ratio (mmHg)	148 (63)	155 (69)	151 (65)
< 100—*n* (%)	15 (31.9%)	9 (27%)	20 (25%)
100–199—*n* (%)	21 (44.7%)	16 (47%)	37 (46%)
200–299—*n* (%)	11 (23.4%)	9 (27%)	24 (30%)
PaO_2_ (mmHg)	84 (25)	84 (27)	84 (26)
FiO_2_	0.64 (0.21)	0.62 (0.25)	0.63 (0.23)
Oxygenation Index (cmH_2_O/mmHg)	13.8 (12.8)	13.3 (11.2)	13.6 (12.1)
mAwP (cmH_2_O)	16.5 (5.4)	17.4 (6.9)	16.9 (6.0)
PEEP (cmH_2_O)	10.4 (4.1)	10.1 (3.3)	10.3 (3.7)
Tidal volume ≤ 8 mL/kg—*n* (%)	32 (68%)	21 (62%)	53 (65%)
PaCO_2_ (mmHg)	42 (16)	44 (13)	43 (15)
pH	7.286 (0.118)	7.263 (0.114)	7.276 (0.116)
Minute ventilation (L/min)	10.8 (4.6)	10.4 (3.3)	10.6 (4.1)
Ventilatory ratio (L mmHg/min kg)	1.9 (1.0)	2.0 (0.8)	1.9 (0.9)
Ventilatory ratio ≥ 2.0—*n* (%)	15 (32%)	11 (32%)	26 (32%)
Additional therapies			
Glucocorticoids—*n* (%)	33 (70%)	25 (74%)	58 (72%)
Neuromuscular blockade—*n* (%)	25 (53%)	19 (56%)	44 (54%)
Pulmonary vasodilators—*n* (%)	6 (13%)	4 (12%)	10 (12%)
Nitric oxide scavengers—*n* (%)	1 (2%)	1 (3%)	2 (3%)
Venovenous-ECMO	2 (4%)	2 (6%)	4 (5%)
Treatment characteristics			
Study drug exposure duration (hrs)—median [IQR]	48 [30, 49]	47 [38, 49]	48 [36, 49]
Mean study drug dose (ng/kg/min)	39 (13)	31 (25)	35 (19)

Baseline characteristics of the study population. Results displayed as mean (SD) unless otherwise indicated

*ACE* angiotensin-converting enzyme, *ARB* angiotensin receptor blockers, *NED* norepinephrine equivalent dose, *ScvO*_*2*_ central venous oxygenation saturation, *PaO*_*2*_ arterial partial pressure of oxygen, *FiO*_*2*_ fraction inspired oxygen, *mAwP* mean airway pressure, *PEEP* positive end-expiratory pressure, *PaCO*_*2*_ arterial partial pressure of carbon dioxide

Groups had similar baseline P:F, OI, and ventilatory ratio. There were no baseline differences in PEEP, mAwP, minute-ventilation, tidal volume, PaCO_2_, pH, or the proportion of patients receiving lung-protective ventilation.

Missing data prevalence is summarized in Additional file 1: Table S1; all missing hour-48 P:F data were due to death before hour-48. The fluid volume administered during the study-drug titration period is shown in Additional file 1: Table S2. The duration of study-drug exposure is shown in Additional file 1: Fig. S2.

### Respiratory measures

**Fig. 1:**
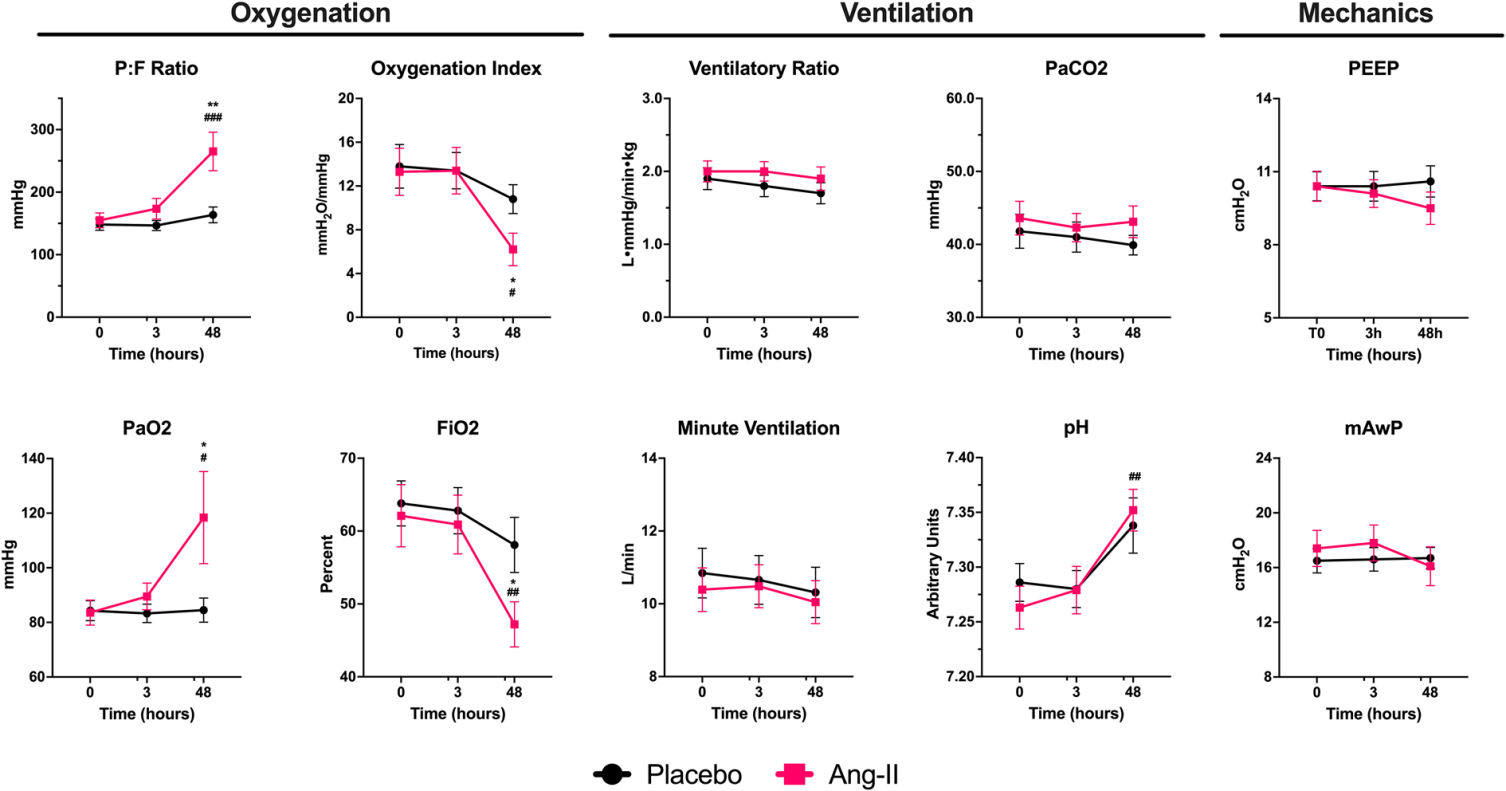
Respiratory Measures Over Time for Angiotensin-II versus Placebo. Displays respiratory variables over time by treatment group. Black indicates the placebo group, pink the Ang-II group. Error bars display the SEM. Asterisks display the p-value for the between-treatment group difference at the indicated timepoint, adjusted for the baseline value, from the regression model as follows: *p < 0.05; **p < 0.01; ***p < 0.001. Hashmarks show the within-treatment difference versus the baseline as follows: ^#^*p* < 0.05; ^##^*p* < 0.01; ^###^*p* < 0.001. *Ang-II* angiotensin-II, *PEEP* positive end-expiratory pressure, *mAwP* mean airway pressure, *SEM* standard error of the mean

Among ARDS patients treated with angiotensin-II, mean P:F increased by 106 mmHg from 155 mmHg (SD: 69) at baseline to 265 mmHg (SD: 160) at hour-48. In contrast, mean P:F did not change (difference: 8 mmHg) in the placebo group (148 mmHg at baseline versus 164 mmHg at hour-48). In the primary analysis model, baseline-adjusted P:F at hour-48 was 98.4 mmHg higher in angiotensin-II patients versus placebo (95%CI: 35.2–161.5 mmHg, *p* = 0.0028) (Table 2 and Fig. 1). The increased P:F at hour-48 in the angiotensin-II group was driven by improvement in both PaO_2_ and FiO_2_, which were 34.4 mmHg higher (95%CI 1.8–66.9 mmHg, *p* = 0.0392) and 10% lower (95%CI − 0.18 to − 0.02, *p* = 0.0200), respectively, than for placebo (Table 2). In the full multivariable model, angiotensin-II treatment was associated with 120.6 mmHg higher P:F at hour-48 than placebo (95%CI 51.0–190.3, *p* = 0.0010) (Additional file 1: Table S3).

**Table 2:** Respiratory measures at hour 48 for angiotensin-II versus placebo

Outcome	Placebo		Angiotensin-II		
	Hour-48	∆ vs. hour-0	Hour-48	∆ vs. hour-0	Baseline-adjusted ∆
	Mean (SD)	Mean (SD) [95%CI] *p*-value	Mean (SD)	Mean (SD) [95%CI] *p*-value	Mean [95%CI] *p*-value
Oxygenation					
P:F (mmHg)	163.6 (73.7)	7.9 (84.2) [− 21.5 to 37.3] *p* = 0.32	265.1 (159.8)	105.9 (172.8) [37.6 to 174.3] *p* = 0.0006	98.4 [35.2 to 161.5] *p* = 0.0028
PaO_2_ (mmHg)	84.5 (25.8)	− 2.4 (30.4) [− 13.0 to 8.3] *p* = 0.98	118.4 (87.6)	34.9 (89.1) [− 0.3 to 70.2] *p* = 0.0322	34.4 [1.8 to 66.9] *p* = 0.0392
FiO_2_	0.58 (0.22)	− 0.02 (0.24) [− 0.11 to 0.06] *p* = 0.24	0.47 (0.16)	− 0.12 (0.19) [− 0.20 to − 0.05] *p* = 0.0092	− 0.10 [− 0.18 to − 0.02] *p* = 0.0200
Oxygenation Index (cmH_2_O/mmHg)	10.8 (7.1)	− 0.4 (8.1) [− 3.6 to 2.7] *p* = 0.26	6.2 (7.0)	− 6.0 (9.2) [− 10.1 to − 1.9] *p* = 0.0118	− 4.8 [− 8.6 to − 1.1] *p* = 0.0121
Ventilation					
Ventilatory ratio	1.8 (0.6)	− 0.2 (0.3) [− 0.35 to 0.02] *p* = 0.42	2.0 (0.7)	− 0.1 (0.4) [− 0.35 to 0.14] *p* = 0.84	0.20 [ − 0.41 to 0.82] *p* = 0.50
PaCO_2_ (mmHg)	39.9 (7.8)	0.2 (8.8) [− 2.9 to 3.3] *p* = 0.51	43.1 (11.3)	− 2.0 (8.8) [− 5.5 to 1.4] *p* = 0.89	0.9 [ − 3.5 to 5.3] *p* = 0.68
pH	7.338 (0.147)	0.023 (0.136) [− 0.020 to 0.070] *p* = 0.0858	7.352 (0.099)	0.059 (0.086) [0.020 to 0.090] *p* = 0.0021	0.010 [− 0.040 to 0.070] *p* = 0.63
Respiratory rate (breaths/minute)	23.9 (7.7)	− 0.3 (6.1) [− 2.29 to 1.71] *p* = 0.91	23.3 (7.9)	− 0.2 (4.1) [− 1.81 to 1.43] *p* = 0.81	− 1.1 [− 3.8 to 1.5] *p* = 0.39
Tidal volume (mL)	425.5 (142.4)	− 2.9 (75.0) [− 28.7 to 22.8] *p* = 0.58	436.5 (151.2)	1.7 (79.7) [− 30.4 to 33.9] *p* = 0.98	16.5 [ − 26.9 to 59.9] *p* = 0.45
Minute ventilation (L/min)	10.31 (4.2)	− 0.46 (2.6) [− 1.36 to 0.43] *p* = 0.59	10.04 (2.95)	− 0.11 (2.49) [− 1.14 to 0.92] *p* = 0.69	− 0.20 [− 1.36 to 0.95] *p* = 0.73
Mechanics					
PEEP (cmH_2_O)	10.6 (3.9)	− 0.1 (2.8) [− 0.99 to 0.89] *p* = 0.86	9.5 (3.5)	− 0.7 (1.9) [− 1.46 to − 0.03] *p* = 0.36	− 0.59 [− 1.61 to 0.43] *p* = 0.26
mAwP (cmH_2_O)	16.7 (4.2)	− 0.4 (3.8) [− 1.90 to 1.09] *p* = 0.85	16.1 (6.2)	− 1.5 (2.4) [− 2.67 to − 0.39] *p* = 0.51	− 1.35 [− 3.07 to 0.38] *p* = 0.12

Displays respiratory physiologic measures at hour-48 in Angiotensin-II vs. placebo. The Hour-48 columns show the average measures within the treatment group. The ∆ vs. hour-0 columns show the average difference at Hour-48 versus baseline within the treatment group, displayed as: Hour-48–Hour-0. The 95% CI and *p*-values in these columns reflect the results of a paired T-test. **T**he baseline-adjusted ∆ column reflects the estimate for difference in means by for Angiotensin-II vs. Placebo groups from the linear model adjusted for the hour-0 value (primary analysis)

∆ difference, *SD *standard deviation, *P:F *PaO_2_/FiO_2_ ratio, *PaO*_*2*_ arterial partial pressure of oxygen, FiO_2_ fraction of inspired oxygen, *mAwP *mean airway pressure, *PEEP* positive end-expiratory pressure, *PaCO*_*2*_ arterial partial pressure of carbon dioxide

Similarly, the OI decreased by hour-48 for the angiotensin-II (-6.0 cmH_2_O/mmHg, SD: 9.2) but not the placebo group (− 0.4 cmH_2_O/mmHg, SD: 8.1) (baseline-adjusted hour-48 difference: − 4.8 cmH_2_O/mmHg, [95%CI − 8.6 to − 1.1], *p* = 0.0273). In the multivariable model (Additional file 1: Table S4), angiotensin-II treatment was numerically but not significantly associated with improved OI at hour-48 (effect-estimate: − 4.2 cmH_2_O/mmHg [95%CI − 8.7 to 0.4], *p* = 0.0694).

In both the baseline-adjusted and multivariable models, VR was similar between groups, suggesting no difference in dead space ventilation (Table 2, Additional file 1: Table S5). There were also no significant differences between treatment groups in PEEP, PaCO_2_, pH, minute-ventilation, lung-protective tidal volume, or mAwP (Fig. 1).

### Cardiovascular measures

**Fig. 2:**
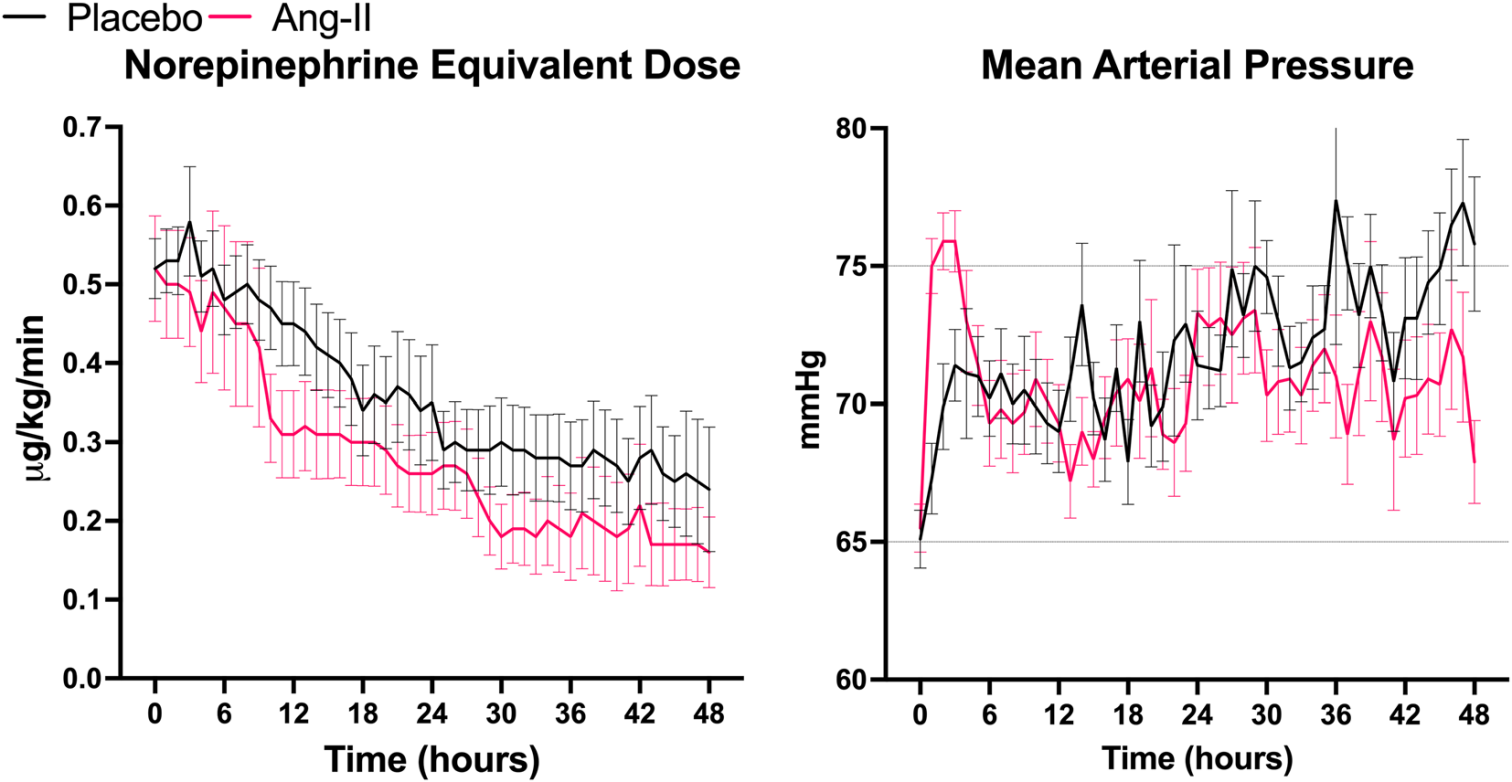
Cardiovascular support measures over time for angiotensin-II versus placebo. Displays the hourly total vasopressor dose in norepinephrine equivalents and mean arterial pressure over time by treatment group. Black curves show the placebo group. Pink curves show the Ang-II group. Error bars display the SEM. *Ang-II* angiotensin-II, *SEM* standard error of the mean

The original ATHOS-3 trial’s primary endpoint, a sustained MAP > 75 mmHg or > 10 mmHg from baseline without an increase in background vasopressor dose at hour-3, was met in 22/34 (64.7%) angiotensin-II and 17/47 (36.2%) placebo patients (absolute risk-difference: 28.5% [95%CI 6.5–47.0], *p* = 0.0112) (Table 3). The average NED over the 48-h study period was significantly lower in the angiotensin-II group (0.28 µg/kg/min) than the placebo group (0.36 µg/kg/min) (difference: 0.08 µg/kg/min [95%CI 0.05–0.10 µg/kg/min], *p* < 0.0001). Differences over time in NED and MAP are shown in Fig. 2.

**Table 3:** Efficacy and exploratory clinical outcomes for angiotensin-II versus placebo

Outcome	Placebo	Angiotensin-II	Absolute difference (CI_95%_)	Relative risk (CI_95%_)	*p*-value
	*n* = 47	*n* = 34			
Hour-3					
Treatment (MAP) response	17 (36.2%)	22 (64.7%)	28.5% (6.5% to 47.0%)	1.79 (1.14 to 2.82)	*p* = 0.0112
Hour-48					
Alive and ventilator-free	1 (2.1%)	0 (0.0%)	− 2.1% (− 12.7% to 10.7%)	0.98 (0.88 to 1.09)	*p* = 0.39
Alive and vasopressor-free	3 (6.4%)	7 (20.6%)	14.2% (− 4.3% to 30.8%)	1.18 (1.00 to 1.49)	*p* = 0.0551
Mortality	14 (29.8%)	7 (20.6%)	− 9.2% (− 30.7% to 9.8%)	0.69 (0.31 to 1.47)	*p* = 0.35
Day-7					
Alive and ventilator-free	5 (10.6%)	7 (20.6%)	10.0% (− 5.8% to 27.2%)	1.94 (0.62 to 6.12)	*p* = 0.21
Alive and vasopressor-free	18 (38.3%)	17 (50.0%)	11.7% (− 9.9% to 32.0%)	1.44 (0.74 to 2.79)	*p* = 0.29
Mortality	25 (53.2%)	14 (41.2%)	− 12.0% (− 32.0% to 9.7%)	0.70 (0.36 to 1.34)	*p* = 0.29
Day-28					
Mortality	32 (68.1%)	19 (55.9%)	− 12.2% (− 32.3% to 8.7%)	0.71 (0.40 to 1.26)	*p* = 0.26
Discharged alive from the hospital	8 (17.0%)	7 (20.6%)	3.6% (− 13.1% to 21.7%)	1.22 (0.44 to 3.37)	*p* = 0.68

Absolute differences and relative risks are reported for the angiotensin-II group versus placebo. Treatment response refers to the primary outcome of the ATHOS-3 trial, which was a MAP either ≥ 75 mmHg or a ≥ 10 mmHg increase from baseline at hour-3

*CI*_*95%*_ 95% Confidence interval, *MAP *mean arterial pressure

### Exploratory vasopressor dose analysis

**Fig. 3:**
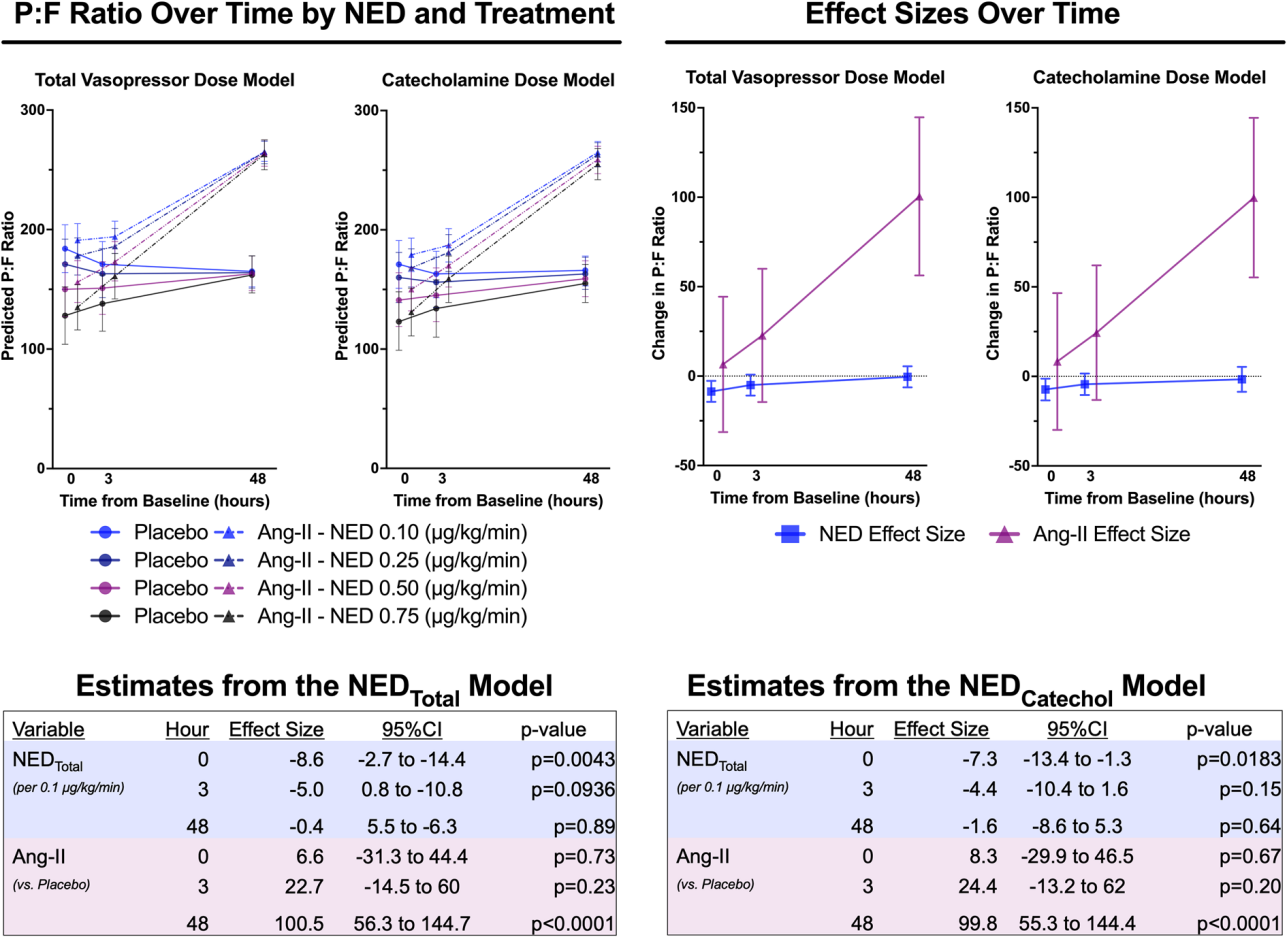
P:F over time by treatment group and vasopressor support level. Left graphs display the predicted P:F over time by treatment group at varying vasopressor dose levels from the longitudinal mixed-effects repeated measures models. Circle markers with solid lines display model estimates for the placebo group. Triangle markers with dashed lines, the angiotensin-II group. Colors are used to display the vasopressor dose: light blue = 0.10 mcg/kg/min; dark blue = 0.25 mcg/kg/min; purple = 0.50 mcg/kg/min; black = 0.75 mcg/kg/min. Right graphs show the model effect size for angiotensin-II treatment (purple triangles) and NED (blue squares) at each time point. Error bars indicate the 95%CIs. The model estimates for both variables are tabulated with 95%CIs and *p*-values at the bottom of the figure. *Ang-II* angiotensin-II, *NED* norepinephrine equivalent dose, *P:F* PaO_2_/FiO_2_ ratio, *95%CI* 95% confidence interval, *NED*_*Total*_ total vasopressor dose in NED, *NED*_*Catechol*_ total catecholamine dose in NED

Figure 3 displays the results of the longitudinal models assessing the total and catecholamine-specific vasopressor dose association with oxygenation between treatment groups. All models estimated no difference in P:F at baseline between angiotensin-II and placebo. P:F between groups began to diverge at hour-3 and was significantly higher in the angiotensin-II group than placebo at hour-48 (Fig. 3). This relationship was preserved across adjustments for both NED_Total_ and NED_Catechol_ (Hour-48 effect size: 100 mmHg and *p* < 0.0001 for both).

In contrast, at baseline, higher NED_Total_ was associated with lower P:F, independent of treatment group (NED_Total_: − 8.6 mmHg per 0.1 µg/kg/min [95%CI: − 14.4 to − 2.7], *p* = 0.0043). The association between NED_Total_ and P:F dissipated over time and was not significant at hour-3 or hour-48 (Hour-48 NED_Total_: − 0.4 mmHg per 0.1 µg/kg/min [95%CI − 6.3–5.5], *p* = 0.89). The same relationship was seen for NED_Catechol_. In summary, adjustment for total or catecholamine-specific vasopressor dose did not attenuate the association of angiotensin-II treatment with improved P:F.

### Exploratory clinical outcomes

Table 3 displays exploratory clinical outcomes. At day-7, the angiotensin-II group had 7/34 (20.6%) patients alive and liberated from the ventilator versus 5/47 (10.6%) placebo patients (difference: 10.0% [95%CI − 5.8 to 27.2%], *p* = 0.21). Similarly, 17/34 (50.0%) patients were alive and off vasopressors in the angiotensin-II group versus 18/47 (38.3%) for placebo (difference: 11.7% [95%CI − 9.9 to 32.0%], *p* = 0.29). By day-28, 19/34 (55.9%) and 32/47 (68.1%) angiotensin-II and placebo patients, respectively, had died while 7/34 (20.6%) and 8/47 (17.0%) were alive and discharged from the hospital.

### Sensitivity analyses

Sensitivity analyses for main study findings are shown in Additional file 1: Tables S6–S10, throughout which, angiotensin-II treatment remained associated with improved oxygenation at hour-48.

## Discussion

In this post-hoc analysis of the ATHOS-3 trial that compared angiotensin-II treatment to placebo in ARDS patients, angiotensin-II was associated with significantly improved oxygenation within 48-h of treatment initiation among patients with ARDS and vasodilatory shock. Moreover, this increase in oxygenation after angiotensin-II treatment was not attributable to measured differences in ventilator management between groups and was independent of catecholamine dose. Finally, twice as many angiotensin-II patients as placebo patients were alive and ventilator-free by day-7, although the study was underpowered to define the significance of this finding.

### Relationship to previous literature

The role of the renin-angiotensin system (RAS) in ARDS remains controversial. Compelling preclinical experiments suggested excess pulmonary AT1R signaling mediates the early development of inflammation-induced and ventilator-induced acute lung injury in rodents [22, 23]. These data led to small trials of RAS antagonism in ARDS and, more recently, larger trials in COVID-19 pneumonia [5, 24–27]. All but one of the COVID-19 studies examined high-dose RAS blockade exclusively within moderate severity disease with very low prevalence of ARDS [25–27]. Therefore, their results cannot be extrapolated to ARDS patients, particularly with concomitant vasodilatory shock. In contrast, the only randomized trial to date of RAS-inhibition in critically-ill COVID-19 patients stopped prematurely for safety [5]. That trial, which compared angiotensin-receptor blockers (ARB) and angiotensin-converting enzyme inhibitors (ACE-i) versus placebo, found > 95% probability of harm from both ARB and ACE-i on organ support-free days, in-hospital mortality, and 90-day survival [5]. Notably, differences in organ support-free days were not driven by vasopressors alone: both RAS-inhibition groups had fewer ventilator-free and respiratory support-free days than placebo (> 90% probability of harm for both). [5]

It is possible that angiotensin-II could benefit patients with ARDS and shock. Several studies in COVID-19 ARDS found improved P:F following angiotensin-II treatment [8–10], although these reports were observational. Another observational study found a similar increase in P:F after angiotensin-II in patients without COVID-19, though this analysis included patients without ARDS [28]. The current analysis demonstrates improved oxygenation after angiotensin-II treatment among non-COVID-19 ARDS patients versus placebo-treated controls.

The mechanism underlying these observations remains uncertain. One possibility is the presence of a relative angiotensin-II deficiency in ARDS. Experimental inflammatory lung injury causes pulmonary endothelial angiotensin-converting enzyme-1 (ACE-1) shedding, which, after transiently increasing systemic angiotensin-II, reduces systemic angiotensin-II levels over time [29, 30]. Several longitudinal biomarker studies report precisely this pattern of RAS dynamics among patients with COVID-19 induced ARDS [31–33]. Small observational studies in non-COVID-19 ARDS also report evidence of endogenous ACE-inhibition [34]. However, why correcting such a deficiency would improve oxygenation is not readily apparent. An anti-inflammatory effect of angiotensin-II elicited through AT2R agonism could be considered, though we note that angiotensin-II has 15-fold higher affinity for AT1R than AT2R [35], making this explanation less likely. Another possibility is that infused angiotensin-II is catalyzed to angiotensin (1–7) by ACE-2, producing anti-inflammatory effects through Mas receptor signaling. Further studies are needed, as the effect of angiotensin-II infusion on non-classical RAS peptide concentrations in ARDS or shock are not known.

Alternatively, or in addition, the relationship between pulmonary vasopressor effects and hypoxic pulmonary vasoconstriction (HPV) may offer insight. Previous literature demonstrates divergent effects of catecholamines and angiotensin-II on the lung vasculature [6, 7]. Norepinephrine, via α_1_-adrenergic signaling, is a potent pulmonary vasoconstrictor that increases total pulmonary vascular resistance (PVR) disproportionately to systemic vascular resistance (SVR) [36]. Unlike angiotensin-II, norepinephrine constricts both the pulmonary arterial and venous systems. [6]

Under normoxic conditions, lung vasculature is more sensitive to angiotensin-II than the systemic circulation [37], where angiotensin-II acts as an arterial-selective pulmonary vasoconstrictor [6]. Hypoxia, however, alters the intrapulmonary effects of angiotensin-II. Cargil et al*.* first reported that alveolar hypoxemia lessens the in vivo vasoconstrictive effects of angiotensin-II in human lung [38]. Similarly, Kiley and colleagues showed, in human volunteers, that during normoxia, AT1R-blockade with losartan did not alter mean pulmonary artery pressure (mPAP) or PVR, but during hypoxemia, losartan reduced both mPAP and PVR [16]. In analogous human experiments, AT1R-inhibition with saralasin inhibited hypoxic vasoconstriction [17]. The improved oxygenation in ARDS after angiotensin-II treatment in the ATHOS-3 study could therefore indicate that angiotensin-II augments HPV, increasing oxygenation by improving V/Q matching.

In contrast to angiotensin-II, catecholamines inhibit HPV, increasing V/Q mismatch. Several studies demonstrate that HPV requires α_1_-adrenergic signaling [18, 39]. These investigations also show that non-specific β-adrenergic blockade heightens the vasoconstrictive response and PVR increase to hypoxemia [39]. Experiments in critically-ill patients and large mammals reported that β-agonists both attenuate HPV and increase shunt fraction. [19, 40, 41]

The interaction of hypoxia and catecholamines raises the possibility that high-dose norepinephrine increases overall pulmonary vasoconstriction while reducing HPV, inhibiting a compensatory mechanism for hypoxemia in ARDS. Sarkar et al*.* showed in porcine hemorrhagic shock that norepinephrine infusion both doubled the PVR/SVR ratio and reduced P:F by 20%. [42]

This literature led us to query whether the difference in oxygenation trajectories in the ARDS subset of ATHOS-3 between angiotensin-II and placebo was attributable to catecholamine dose-reduction. Supporting this hypothesis, placebo group oxygenation was substantially lower at hour-48, when these patients were receiving 50% higher NED (0.24 µg/kg/min) than the angiotensin-II group (0.16 µg/kg/min). At hour-3, when oxygenation was more similar between groups, placebo group NED (0.58 µg/kg/min) was only 18% higher than the angiotensin-II group (0.49 µg/kg/min) and both groups remained on high catecholamine doses. However, when we analyzed the associations of angiotensin-II treatment and longitudinal vasopressor dose with oxygenation, we found angiotensin-II was robustly associated with improved P:F independent of NED, whereas NED was not significantly associated with P:F. We found identical results in sensitivity analysis of the catecholamine-specific NED. These results are consistent with a direct effect of angiotensin-II to increase oxygenation, and not indirect effects mediated through catecholamine dose reduction.

However, while this analysis suggests a direct effect of angiotensin-II, it does not necessarily implicate HPV. Extrapolating from the nitric oxide literature, improving V/Q matching does not appear inherently disease modifying in ARDS [43, 44]. Improved oxygenation in the treatment arm could alternatively relate to the complex effects of the RAAS on immune regulation [45, 46], or simply represent an epiphenomenon of better treatment of shock.

### Implications of study findings

Our study findings imply angiotensin-II improves oxygenation to a clinically relevant degree in patients with ARDS and vasodilatory shock. Prospective clinical trials investigating angiotensin-II as a treatment in ARDS are now needed to validate or refute these findings.

Moreover, these data suggest enhanced oxygenation with angiotensin-II treatment more likely reflects a direct effect of angiotensin-II rather than an effect of catecholamine dose reduction. Thus, our study highlights a need for renewed physiologic investigation into the effect of vasoactive therapies on respiratory gas exchange in ARDS.

Finally, we note that a previous criticism of the ATHOS-3 trial had been the higher prevalence of ARDS in the placebo group [47]. We now show that in that subset of ATHOS-3 patients with ARDS at enrollment, angiotensin-II met the trial’s primary efficacy endpoint and was associated with improved oxygenation. Therefore, higher ARDS prevalence in the control arm of the overall ATHOS-3 cohort cannot explain the favorable outcomes found in the trial’s primary and pre-specified analyses [1, 48]. At a minimum, angiotensin-II appears to be a safe treatment in patients with catecholamine-refractory vasodilatory shock with concomitant ARDS.

### Strengths and limitations

The multicenter international structure, randomized treatment allocation, placebo-comparator, and double-blinded design of the ATHOS-3 trial all increase our confidence in these results. However, we also stress that *post-hoc* subgroup analyses cannot confirm causal effects of treatment [49]. Additionally, increased oxygenation following angiotensin-II infusion was not attributable to differences in ventilator management or baseline severity-of-illness. Initial vasopressor requirements, hemodynamic and respiratory parameters, and APACHE-II scores were well balanced between treatment groups. There was no difference between treatment groups in PEEP, minute-ventilation, or implementation of lung-protective tidal volumes that would explain improved oxygenation among only the angiotensin-treated patients. The similar levels of PEEP during the study period are particularly important: more robust systemic hemodynamics could contribute to improved oxygenation if they facilitated more aggressive PEEP titration and alveolar recruitment. This potential bias seems unlikely in the absence of a difference in PEEP management between groups.

However, several limitations impact this study. First, fluid balance, filling pressures, and serial echocardiography were not measured. The placebo group could have failed to improve oxygenation if they more often developed volume overload. Second, this was a *post-hoc* analysis of 81 patients which incurs the risk of small-sample bias and other type-I errors [49]. Third, the primary trial protocol was focused on refractory shock and did not protocolize respiratory management. All included patients met criteria for ARDS at baseline but 35% of patients were not receiving low tidal volume ventilation at that time. The frequency of low tidal volume ventilation was similar between treatment groups suggesting this factor does not confound our results, but findings could differ in a population where a higher proportion of patients received lung-protective management. Relatedly, PEEP titration and lung recruitment were also not protocolized, and data on the strategies and timing used for these factors were not available. Data on the ventilation strategy, (e.g., assisted vs. controlled) were also not available). Fourth, collecting respiratory data at hour-3 and hour-48 leaves a significant gap that could coincide with a dynamic part of our subjects’ respiratory course. It was also not known how early or late in an ARDS course patients were. Fifth, VR is an imperfect surrogate for dead space ventilation [15]. Sixth, we applied modified Berlin criteria to select patients and cannot exclude that some patients may have had a fluid overload component of their hypoxemia at baseline. Seventh, prone positioning is an important intervention for severe refractory hypoxemia in ARDS, but data on its implementation were not available. Eighth, this analysis was underpowered to assess patient outcomes in the ARDS subset and we cannot attribute differences in these endpoints to the effects of treatment. Still, whether due to chance or other causes, the fact that twice as many angiotensin-treated as placebo-treated patients were alive and ventilator-free on day-7 provides reassurance that the physiological effects seen were not dissociated from clinical outcomes.

## Conclusion

In post-hoc subgroup analysis of an international, multicenter, double-blind randomized trial among patients with ARDS and catecholamine-refractory distributive shock, angiotensin-II was associated with improved oxygenation within 48-h of therapy versus placebo independent of ventilator management. Targeted, experimental studies are now needed in ARDS to determine the mechanisms driving this observation. These findings additionally provide a physiological rationale for prospective clinical trials testing angiotensin-II as a treatment in ARDS.

### Supplementary Information

**Additional file 1: Figure S1. **DAG for Direct vs. Indirect Effect of Ang-II on Oxygenation. **Figure S2**. Duration of Study Drug Exposure. **Table S1**: Prevalence of Missing Data. **Table S2.** Fluid Administration During Study Drug Titration Period. **Table S3**. Full Multivariable Model for PaO2/FiO2 Ratio at Hour 48. **Table S4.** Full Multivariable Model for Oxygenation Index at Hour 48. **Table S5**. Full Multivariable Model for Ventilatory Ratio at Hour 48. **Table S6**. Sensitivity Analyses Using Different Missing Data Strategies. **Table S7.** Sensitivity Analyses Excluding Patients with Prior ACEi/ARB Exposure. **Table S8.** Model for PaO2/FiO2 Ratio at Hour 48 Adjusting for ACEi/ARB Exposure. **Table S9**. Sensitivity Analyses Excluding Patients with ECMO Exposure. **Table S10**. PaO2/FiO2 Ratio at Hour 48 According to Baseline Hypoxemia Severity.

## Data Availability

The data that support the findings of this study were used under license from La Jolla Pharmaceutical Company for the current study. Data are available from the authors upon reasonable request and with permission of La Jolla Pharmaceutical Company.

## Abbreviations

ARDS: Acute respiratory distress syndrome
ACE-i: Angiotensin-converting enzyme inhibitors
ARB: Angiotensin receptor blockers
APACHE-II: Acute physiology and Chronic Health Evaluation II score
AT1R: Angiotensin-II Type-1 Receptor
ATHOS-3: Angiotensin-II for the Treatment of High-Output Shock-3 (ATHOS-3) trial
HPV: Hypoxic pulmonary vasoconstriction
MAP: Mean arterial pressure
mAwP: Mean airway pressure
NED: Norepinephrine equivalent dose (in µg/kg/min)
OI: Oxygenation index
P:F: PaO_2_/FiO_2_ ratio
PEEP: Positive end-expiratory pressure
RAS: Renin-angiotensin system
VR: Ventilatory ratio
