# Supplementary material for: Angiotensin II treatment is associated with improved oxygenation in ARDS patients with refractory vasodilatory shock
Source: Ann Intensive Care. 2023 Dec 16;13:128. doi: 10.1186/s13613-023-01227-5 (PMC10725390; doi:10.1186/s13613-023-01227-5)
Supplement: Supplementary file 1 — Additional file 1: Figure S1. DAG for Direct vs. Indirect Effect of Ang-II on Oxygenation. Figure S2. Duration of Study Drug Exposure. Table S1: Prevalence of Missing Data. Table S2. Fluid Administration During Study Drug Titration Period. Table S3. Full Multivariable Model for PaO2/FiO2 Ratio at Hour 48. Table S4. Full Multivariable Model for Oxygenation Index at Hour 48. Table S5. Full Multivariable Model for Ventilatory Ratio at Hour 48. Table S6. Sensitivity Analyses Using Different Missing Data Strategies. Table S7. Sensitivity Analyses Excluding Patients with Prior ACEi/ARB Exposure. Table S8. Model for PaO2/FiO2 Ratio at Hour 48 Adjusting for ACEi/ARB Exposure. Table S9. Sensitivity Analyses Excluding Patients with ECMO Exposure. Table S10. PaO2/FiO2 Ratio at Hour 48 According to Baseline Hypoxemia Severity. [file 13613_2023_1227_MOESM1_ESM.pdf]

Supplemental Materials

Angiotensin II Treatment is Associated with Improved Oxygenation in ARDS Patients with Refractory Vasodilatory Shock

Leisman D, Handisides D, Chawla L, *et al*.

Table of Contents:

1. **Supplemental Methods**
   a. Missing Data Management - pg. s1
   b. Additional Sensitivity and Subgroup Analyses - pg. s1

2. **Supplemental Figures**
   a. **Figure S1:** DAG for Direct vs. Indirect Effect of Ang-II on Oxygenation - pg. s3
   b. **Figure S2:** Duration of Study Drug Exposure - pg. s4

3. **Supplemental Tables:**
   a. **S1**: Prevalence of Missing Data - pg. s5
   b. **S2**: Fluid Administration During Study Drug Titration Period - pg. s6
   c. **S3**: Full Multivariable Model for PaO_2/FiO_2 Ratio at Hour 48 - pg. s7
   d. **S4**: Full Multivariable Model for Oxygenation Index at Hour 48 - pg. s8
   e. **S5**: Full Multivariable Model for Ventilatory Ratio at Hour 48 - pg. s9
   f. **S6**: Sensitivity Analyses Using Different Missing Data Strategies - pg. s10
   g. **S7**: Sensitivity Analyses Excluding Patients with Prior ACEi/ARB Exposure - pg. s11
   h. **S8**: Model for PaO_2/FiO_2 Ratio at Hour 48 Adjusting for ACEi/ARB Exposure - pg. s12
   i. **S9**: Sensitivity Analyses Excluding Patients with ECMO Exposure - pg. s13
   j. **S10**: PaO_2/FiO_2 Ratio at Hour 48 According to Baseline Hypoxemia Severity - pg. s14

SUPPLEMENTAL METHODS

Missing Data Management

For the primary analysis of respiratory measures at hour-48, we used complete-case analysis. This method was selected instead of multiple imputation because imputation assumes data to be missing at random and can be more prone to producing biased estimates when this assumption is violated.[1] In this study, all patients with missing hour-48 P:F were missing this data because they died prior to that timepoint, and therefore the data were not randomly missing. We took several steps to ensure analysis were not distorted by subject attrition. First, the mixed-effects models used in the vasopressor dose analysis provide a validity check, as these models are robust to attrition bias.[2]

Next, we performed two additional sensitivity analyses under optimistic and pessimistic assumptions. For the first of these, we re-performed the primary analysis using last-observation carried forward (LOCF) for the patients that died before hour-48. LOCF likely assigns optimistic values because it may attribute a less severely abnormal value to a patient that subsequently had a poor outcome. The pessimistic sensitivity analysis used worst-case values, where patients that died before outcome measurement were assigned an extreme value for the hour-48 time point. These were P:F=50, PaO2=50, FiO2 = 100%, OI = 50, and VR = 4.0, respectively. This approach may attribute overly pessimistic values to patients that may have had less severe respiratory dysfunction had they survived to hour-48.

We reasoned that while all four approaches – i.e., complete-case, mixed-effects models, LOCF, and worst-case values – all have potential advantages and disadvantages, concordant results between them would suggest that attrition bias was unlikely to be affected our study’s results.

Additional Sensitivity and Subgroup Analyses

As additional sensitivity analyses to account for prior ACE-inhibitor or ARB exposure, we recapitulated both when excluding these patients as well as by adding a covariate the multivariable

model for ACE-inhibitor/ARB exposure. We similarly performed sensitivity analysis excluding patients that received extracorporeal membrane oxygenation. Finally, as a post-hoc exploratory analysis, we assessed whether the effect of angiotensin-II on the primary outcome of hour-48 P:F varied by baseline hypoxemia severity. We first recapitulated the primary analysis, stratifying patients based on whether baseline hypoxemia was mild (P:F 200-299), moderate (P:F 100-199), or severe (P:F <100). We next analyzed the baseline P:F continuously by including an interaction term for baseline P:F and treatment group in the model.

SUPPLEMENTAL FIGURES

Figure S1: Directed Acyclic Graph for Direct vs. Indirect Effect of Angiotensin-II on Oxygenation Hypotheses

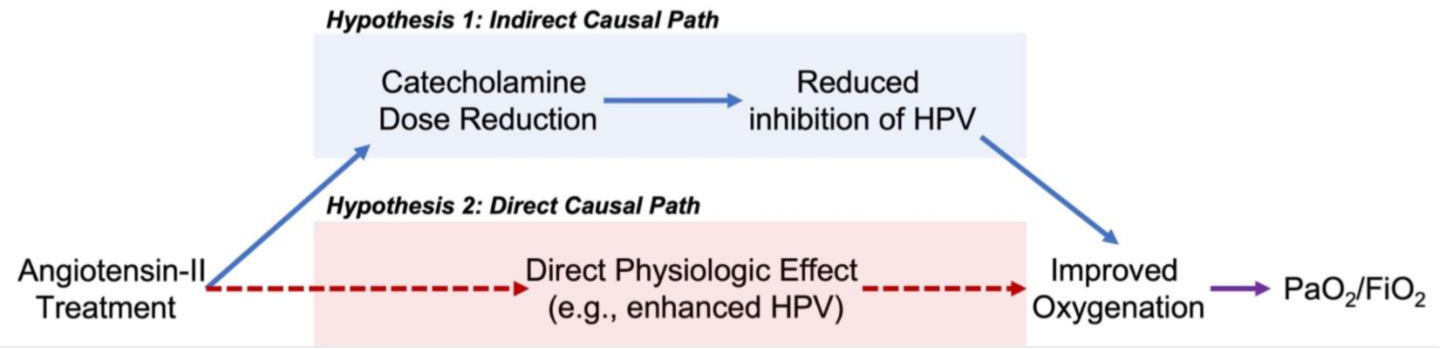

Legend: Directed acyclic graph showing hypothesized causal pathway for a direct vs. indirect effect of angiotensin-II treatment on oxygenation.

Figure S2: Duration of Study Drug Exposure

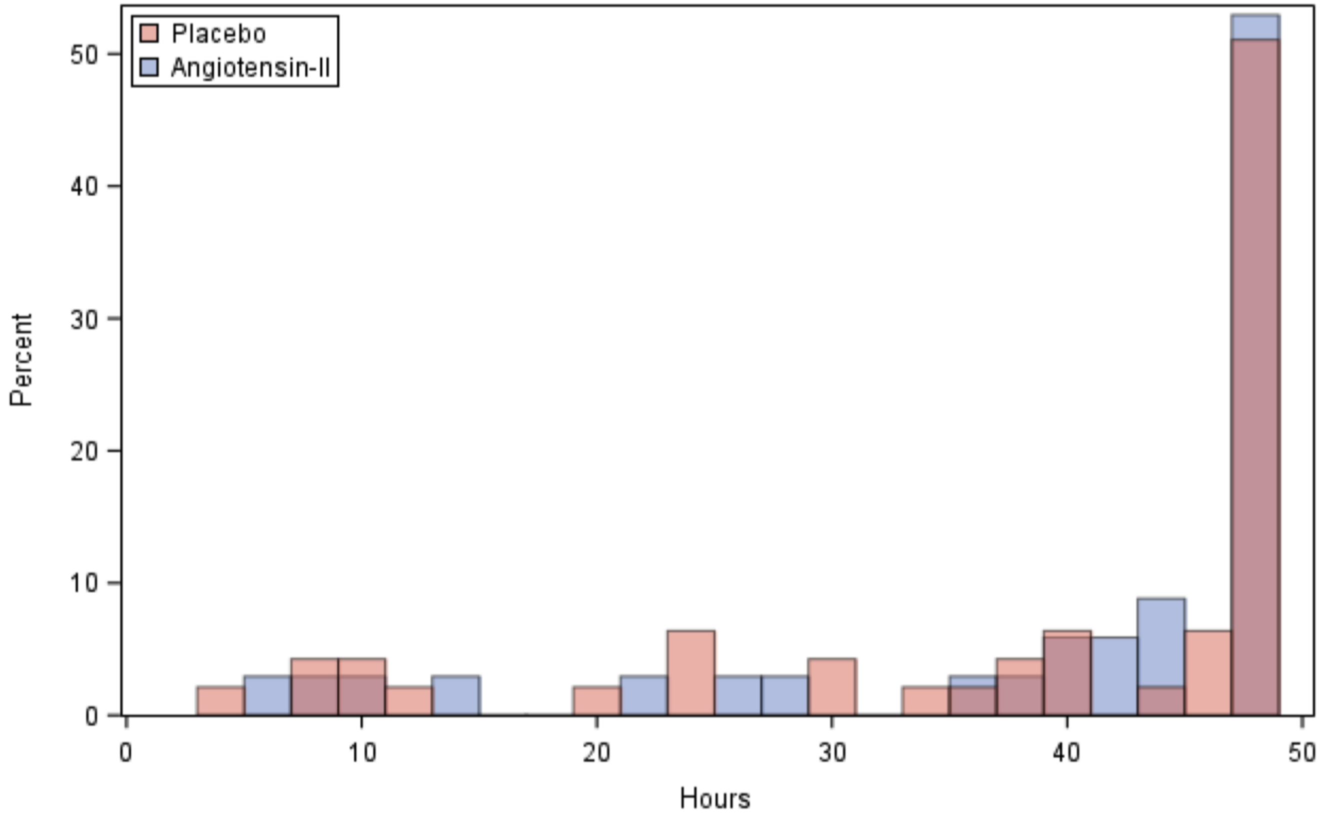

Legend: Histogram showing study drug exposure in hours by treatment group. In the graph, patients with study drug exposure greater than 48-hours are binned together as >48hours.

SUPPLEMENTAL TABLES

Table S1: Prevalence of Missing Data

Outcome	Placebo	Angiotensin-II	All Patients
**N**	47	*34*	*81*
**Demographics and Clinical Factors**			
Age	0	0	0
Female	0	0	0
Body Mass Index	1 (2%)	0	1 (1%)
Ideal Body Weight	1 (2%)	0	1 (1%)
Exposure to ACE inhibitors or ARBs	0	0	0
Cause of Vasodilatory Shock	0	0	0
Baseline APACHE II Score	0	0	0
Baseline Albumin	1 (2%)	3 (9%)	4 (5%)
**Baseline Cardiovascular Status**			
Mean Arterial Pressure	0	0	0
Average NED in past 6 hours	1 (2%)	0	1 (1%)
Vasopressin use in past 6 hours	0	0	0
Central Venous Pressure	14 (30%)	6 (18%)	20 (25%)
Cardiac Index	26 (55%)	17 (50%)	43 (53%)
$ScvO_2$	14 (30%)	8 (24%)	22 (27%)
**Baseline Respiratory Status**			
PaO_2:FiO_2 ratio	0	0	0
PaO_2	0	0	0
FiO_2	0	0	0
Oxygenation Index	10 (21%)	7 (21%)	17 (21%)
mAwP	10 (21%)	7 (21%)	17 (21%)
PEEP	1 (2%)	0	1 (1%)
Tidal Volume	1 (2%)	2 (6%)	3 (4%)
$PaCO_2$	0	0	0
pH	0	0	0
Minute Ventilation	1 (2%)	3 (9%)	4 (5%)
Ventilatory Ratio	1 (2%)	2 (6%)	3 (4%)
**Additional Therapies**			
Glucocorticoids	0	0	0
Neuromuscular Blockade	0	0	0
Pulmonary Vasodilators – n (%)	0	0	0
Nitric Oxide Scavengers – n (%)	0	0	0
Venovenous-ECMO	0	0	0
**Treatment Characteristics**			
Duration of Study Drug Exposure	0	0	0
Mean Study Drug Dose	0	0	0
**Hour-48 Outcomes**			
PaO_2:FiO_2 ratio	13 (28%)	7 (21%)	20 (25%)
PaO_2	13 (28%)	7 (21%)	20 (25%)
FiO_2	13 (28%)	7 (21%)	20 (25%)
Oxygenation Index	23 (49%)	18 (53%)	41 (51%)
mAwP	18 (38%)	15 (44%)	33 (41%)
PEEP	10 (21%)	6 (18%)	16 (20%)
Tidal Volume	11 (23%)	8 (24%)	19 (23%)
$PaCO_2$	13 (28%)	7 (21%)	20 (25%)
pH	13 (28%)	7 (21%)	20 (25%)
Minute Ventilation	11 (23%)	9 (26%)	20 (25%)
Ventilatory Ratio	19 (40%)	12 (35%)	31 (38%)

All results displayed as number of subjects (%) missing. Abbreviations: ACE – angiotensin converting enzyme; ARB – angiotensin receptor blockers; NED – norepinephrine equivalent dose; $ScvO_2$ – central venous oxygenation saturation; PaO_2 – arterial partial pressure of oxygen; FiO_2 – fraction inspired oxygen; mAwP – mean airway pressure; PEEP – positive end-expiratory pressure; $PaCO_2$ – arterial partial pressure of carbon dioxide; ECMO – extracorporeal mechanical oxygenation.

Table S2: Intravenous Fluid Administration During Study Drug Titration Period in Angiotensin-II versus Placebo

Placebo	Angiotensin-II	Difference	95% Confidence Interval	p-value
678 mL (SD: 456)	425 mL (SD: 290)	-254 mL	-419 to -88 mL	p=0.0031

Displays the mean fluid volumes and differences between groups in administered fluid volumes during the study drug titration period. The 95% confidence interval and p-value were calculated using an unpaired T-test with Welch's correction for unequal variances.

Table S3: Full Multivariable Model for PaO_2/FiO_2 Ratio at Hour 48

Parameter	Effect Estimate	95% CI	p value
**Angiotensin-II vs. Placebo**	**98.1**	**26.2 to 170.0**	**p=0.0086**
Baseline P:F Ratio (per mmHg)	0.2	-0.5 to 0.9	p=0.57
Age (per 10 years)	-8.2	-3.2 to 1.5	p=0.48
Female Sex	44.3	-36.6 to 125.1	p=0.28
Body Mass Index (per kg/m2)	3.2	-1.6 to 7.9	p=0.18
Baseline MAP (per mmHg)	0.8	-5 to 6.7	p=0.77
Baseline NED (per 0.1 mcg/kg/min)	78.8	-63.3 to 220.9	p=0.27
Baseline APACHE-II	-2.1	-6.7 to 2.5	p=0.37
Baseline PEEP (per cmH2O)	-1.3	-11.4 to 8.7	p=0.79
Baseline Minute Ventilation (per mL/min)	0.1	-0.5 to 0.8	p=0.68

Full output for the multivariable fixed-effects model for P:F Ratio at hour-48. Effect estimate refers to the change in P:F Ratio associated with a 1-unit increase in the indicated continuous variable or vs. the reference group for binary variables.
Abbreviations: P:F Ratio – PaO2/FiO2 Ratio; MAP – mean arterial pressure; NED – norepinephrine equivalent dose; APACHE-II – acute physiology and chronic health II score; PEEP – positive end-expiratory pressure.

Table S4: Full Multivariable Model for Oxygenation Index at Hour 48

Parameter	Effect Estimate	95% CI	p value
**Angiotensin-II vs. Placebo**	**-4.2**	**-8.7 to 0.4**	**p=0.0694**
Baseline Oxygenation Index (per cmH2O/mmHg)	0.4	0.1 to 0.6	p=0.0045
Age (per 10 years)	0.9	-0.6 to 2.4	p=0.23
Female Sex	0.2	-4.9 to 5.2	p=0.95
Body Mass Index (per kg/m2)	0.3	0.0 to 0.6	p=0.0563
Baseline MAP (per mmHg)	1.8	-1.9 to 5.5	p=0.32
Baseline NED (per 0.1 mcg/kg/min)	0.1	-0.80 to 1.0	p=0.89
Baseline Apache-II	0.0	-0.3 to 0.3	p=0.95
Baseline PEEP (per cmH2O)	-0.1	-0.7 to 0.5	p=0.70
Baseline Minute Ventilation (per mL/min)	0.0	-0.1 to 0.0	p=0.28

Full output for the multivariable fixed-effects model for oxygenation index at hour-48. Effect estimate refers to the change in P:F Ratio associated with a 1-unit increase in the indicated continuous variable or vs. the reference group for binary variables.
Abbreviations: MAP – mean arterial pressure; NED – norepinephrine equivalent dose; APACHE-II – acute physiology and chronic health II score; PEEP – positive end-expiratory pressure.

Table S5: Full Multivariable Model for Ventilatory Ratio at Hour 48

Parameter	Effect Estimate	95%CI	p value
**Angiotensin-II vs. Placebo**	**-0.02**	**-0.35 to 0.32**	**p=0.92**
Baseline Ventilatory Ratio (per L•mmHg/min•kg)	0.81	0.51 to 1.10	p<0.0001
Age (per 10 years)	0.01	-0.01 to 0.02	p=0.29
Female Sex	-0.21	-0.58 to 0.16	p=0.26
Body Mass Index (per kg/m2)	0.00	-0.03 to 0.02	p=0.66
Baseline MAP (per mmHg)	-0.01	-0.03 to 0.02	p=0.63
Baseline NED (per 0.1 mcg/kg/min)	-0.22	-0.83 to 0.40	p=0.47
Baseline Apache-II	-0.01	-0.03 to 0.01	p=0.53
Baseline PEEP (per cmH2O)	-0.02	-0.06 to 0.03	p=0.46
Baseline Minute Ventilation (per mL/min)	0.00	0.00 to 0.00	p=0.87

Full output for the multivariable fixed-effects model for ventilatory ratio at hour-48. Effect estimate refers to the change in P:F Ratio associated with a 1-unit increase in the indicated continuous variable or vs. the reference group for binary variables.
Abbreviations: MAP – mean arterial pressure; NED – norepinephrine equivalent dose; APACHE-II – acute physiology and chronic health II score; PEEP – positive end-expiratory pressure.

Table S6: Sensitivity Analyses Comparing Respiratory Outcome Measures at Hour-48 With Different Missing Data Management Approaches

Outcome	Placebo		Angiotensin-II		
	Hour-48 Mean (SD)	Δ vs. Hour-0 Mean (SD) [95%CI] p-value	Hour-48 Mean (SD)	Δ vs. Hour-0 Mean (SD) [95%CI] p-value	Baseline-Adjusted Δ Mean [95%CI] p-value
**Complete Case**					
P:F (mmHg)	163.6 (73.7)	7.9 (84.2) [-21.5 to 37.3] p=0.32	265.1 (159.8)	105.9 (172.8) [37.6 to 174.3] p=0.0006	**+98.4 [35.2 to 161.5] p=0.0028**
PaO_2 (mmHg)	84.5 (25.8)	-2.4 (30.4) [-13.0 to 8.3] p=0.98	118.4 (87.6)	34.9 (89.1) [-0.3 to 70.2] p=0.0322	**+34.4 [1.8 to 66.9] p=0.0392**
FiO_2	0.58 (0.22)	-0.02 (0.24) [-0.11 to 0.06] p=0.24	0.47 (0.16)	-0.12 (0.19) [-0.20 to -0.05] p=0.0092	**-0.10 [-0.18 to -0.02] p=0.0200**
Oxygenation Index (cmH_2O/mmHg)	10.8 (7.1)	-0.4 (8.1) [-3.6 to 2.7] p=0.26	6.2 (7.0)	-6.0 (9.2) [-10.1 to -1.9] p=0.0118	**-4.8 [-8.6 to -1.1] p=0.0121**
Ventilatory Ratio	1.8 (0.6)	-0.2 (0.3) [-0.4 to 0.0] p=0.42	2.0 (0.7)	-0.1 (0.4) [-0.4 to 0.1] p=0.84	**+0.2 [-0.4 to 0.8] p=0.50**
**Last Observation Carried Forward**					
P:F (mmHg)	152.9 (73.5)	4.6 (72.7) [-16.8 to 25.9] p=0.75	235.7 (154.8)	80.8 (162.1) [24.2 to 137.4] p=0.0070	**+80.3 [29.8 to 130.7] p=0.0022**
PaO_2 (mmHg)	82.2 (24.9)	-2.1 (28.5) [-10.5 to 6.3] p=0.68	111.2 (79.4)	27.6 (81.0) [-0.7 to 55.8] p=0.0592	**+29.2 [4.8 to 53.6] p=0.0195**
FiO_2	0.62 (0.23)	-0.02 (0.21) [-0.08 to 0.04] p=0.68	0.53 (0.22)	-0.09 (0.19) [-0.16 to -0.02] p=0.12	**-0.07 [-0.16 to 0.03] p=0.0580**
Oxygenation Index (cmH_2O/mmHg)	12.9 (11.2)	-0.9 (7.3) [-3.2 to 1.5] p=0.74	-9.0 (8.9)	-4.3 (8.6) [-7.6 to -1.2] p=0.10	**-3.7 [-6.9 to -0.4] p=0.0268**
Ventilatory Ratio	1.8 (1.0)	0.0 (0.4) [-0.1 to 0.1] p=0.95	2.0 (0.7)	0.0 (0.5) [-0.2 to 0.2] p=0.93	**0.0 [-0.2 to 0.2] p=0.92**
**Worst Case Value**					
P:F (mmHg)	132.1 (80.8)	-16.2 (92.0) [-43.2 to 10.8] p=0.28	220.8 (167.1)	65.9 (175.8) [4.5 to 127.2] p=0.0373	**+87.2 [31.4 to 143.0] p=0.0026**
PaO_2 (mmHg)	75.0 (26.9)	-9.4 (32.5) [-18.9 to 0.2] p=0.0833	104.3 (82.6)	20.7 (84.5) [-8.8 to 50.2] p=0.17	**+29.5 [3.9 to 55.2] p=0.0246**
FiO_2	0.70 (0.27)	0.06 (0.27) [-0.02 to 0.14] p=0.24	0.58 (0.26)	-0.04 (0.27) [-0.13 to 0.05] p=0.52	**-0.11 [-0.22 to 0.00] p=0.0527**
Oxygenation Index (cmH_2O/mmHg)	22.9 (19.3)	8.5 (18.1) [2.5 to 14.5] p=0.0128	17.9 (20.6)	4.6 (20.4) [-3.0 to -12.2] p=0.2883	**-4.2 [-13.3 to 4.8] p=0.36**
Ventilatory Ratio	2.5 (1.3)	0.6 (1.3) [0.3 to 1.0] p=0.0112	2.5 (1.1)	0.5 (1.1) [0.2 to 0.9] p=0.0293	**0.0 [-0.5 to 0.6] p=0.92**

Respiratory physiologic measures at hour-48 in angiotensin-II vs. placebo under different missing data management strategies. The Hour-48 columns show the average measures within the treatment group. The Δ vs. hour-0 columns show the mean difference at Hour-48 vs. baseline within the treatment group, displayed as: Hour-48 – Hour-0. The baseline-adjusted Δ column reflects the estimate for difference in means by for angiotensin-II vs. placebo groups from the linear model adjusted for the hour-0 value (primary analysis).
Abbreviations: Δ – difference; SD – standard deviation; P:F – PaO_2/FiO_2 Ratio; PaO_2 – arterial partial pressure of oxygen; FiO_2 – fraction of inspired oxygen; mAwP – mean airway pressure; PEEP – positive end-expiratory pressure; $PaCO_2$ – arterial partial pressure of carbon dioxide.

Table S7: Sensitivity Analyses For PaO_2/FiO_2 Ratio at Hour-48 Excluding Patients With Prior Exposure to ARBs or ACE-Inhibitors (All Missing Data Approaches)

Outcome	Placebo		Angiotensin-II		
	Hour-48 Mean (SD)	Δ vs. Hour-0 Mean (SD) [95%CI] p-value	Hour-48 Mean (SD)	Δ vs. Hour-0 Mean (SD) [95%CI] p-value	Baseline-Adjusted Δ Mean [95%CI] p-value
**Complete Case**	166.2 (76.6)	9.6 (86.1) [-22.0 to 41.8] p=0.32	256.5 (162.0)	95.7 (178.7) [20.2 to 171.1] p=0.0006	**90.1 [23.3 to 156.9] p=0.0092**
**Last Observation Carried Forward**	154.0 (75.7)	5.5 (73.5) [-16.9 to 27.9] p=0.71	238.7 (157.0)	77.0 (171.7) [10.5 to 143.6] p=0.0216	**-80.6 [25.8 to 135.4] p=0.0045**
**Worst Case Value**	131.9 (83.5)	-16.6 (93.8) [-45.2 to 11.9] p=0.30	227.0 (166.6)	65.3 (183.9) [-6.0 to 136.6] p=0.0618	**93.7 [34.0 to 153.3] p=0.0025**

Displays P:F at hour-48 in angiotensin-II vs. placebo when excluding patients with prior exposure to ARBs or ACE-inhibitors under all missing data management strategies. The Hour-48 columns show the average P:F within the treatment group. The Δ vs. hour-0 columns show the mean difference at Hour-48 vs. baseline within the treatment group, displayed as: Hour-48 – Hour-0. The 95% CI and p-values in these columns reflect the results of a paired T-test. The baseline-adjusted Δ column reflects the estimate for difference in means by for Angiotensin-II vs. Placebo groups from the linear model adjusted for the hour-0 value (primary analysis).
Abbreviations: Δ – difference; SD – standard deviation; P:F – PaO_2/FiO_2 Ratio; ARB – angiotensin receptor blocker; ACE – angiotensin converting enzyme; 95%CI – 95% confidence interval.

Table S8: Sensitivity Analysis - Full Multivariable Model for PaO_2/FiO_2 Ratio at Hour 48 Including Adjustment for Prior ARB and ACE-Inhibitor Exposure

Parameter	Effect Estimate	95%CI	p value
**Angiotensin-II vs. Placebo**	**98.5**	**26.1 to 171.0**	**p=0.0088**
Baseline P:F Ratio (per mmHg)	0.2	-0.5 to 0.9	p=0.61
Age (per 10 years)	-9.2	-33.1 to 14.6	p=0.44
Female Sex	44.0	-37.4 to 125.5	p=0.28
Body Mass Index (per kg/m2)	3.1	-1.7 to 7.8	p=0.20
Baseline MAP (per mmHg)	0.6	-5.4 to 6.6	p=0.85
Baseline NED (per 0.1 mcg/kg/min)	7.0	-7.6 to 21.6	p=0.34
Baseline APACHE-II	-2.1	-6.7 to 2.5	p=0.36
Baseline PEEP (per cmH2O)	-1.8	-12.0 to 8.4	p=0.73
Baseline Minute Ventilation (per mL/min)	0.1	-0.5 to 0.8	p=0.72
Prior ARB/ACEi Exposure	30.5	-66.4 to 127.4	p=0.53

Full output for the sensitivity analysis multivariable fixed-effects model for P:F Ratio at hour-48 that also adjusted for prior ARB/ACEi exposure. Effect estimate refers to the change in P:F Ratio associated with a 1-unit increase in the indicated continuous variable unless otherwise indicated or the change vs. the reference group for binary variables.
Abbreviations: P:F Ratio – PaO2/FiO2 Ratio; MAP – mean arterial pressure; NED – norepinephrine equivalent dose; APACHE-II – acute physiology and chronic health II score; PEEP – positive end-expiratory pressure; ARB – angiotensin receptor blocker; ACEi – Angiotensin Converting Enzyme Inhibitor; 95%CI – 95% confidence interval.

Table S9: Sensitivity Analyses For PaO_2/FiO_2 Ratio Excluding Patients Who Received ECMO During Hospitalization (All Missing Data Approaches)

Outcome	Placebo: Hour-48 Mean (SD)	Placebo: Δ vs. Hour-0 Mean (SD) [95%CI] p-value	Angiotensin-II: Hour-48 Mean (SD)	Angiotensin-II: Δ vs. Hour-0 Mean (SD) [95%CI] p-value	Baseline-Adjusted Δ Mean [95%CI] p-value
Complete Case	163.4 (71.3)	9.6 (78.7) [-18.8 to 38.0] p=0.32	239.1 (115.6)	80.4 (115.0) [32.9 to 127.8] p=0.0006	73.6 [24.9 to 122.3] p=0.0037
Last Observation Carried Forward	152.3 (71.7)	5.6 (67.7) [-14.8 to 26.0] p=0.70	213.6 (114.9)	59.3 (110.1) [19.6 to 99.0] p=0.0138	56.8 [17.8 to 95.7] p=0.0048
Worst Case Value	130.6 (79.3)	-16.0 (89.0) [-42.8 to 10.7] p=0.29	197.7 (129.0)	43.4 (127.9) [-2.7 to 89.5] p=0.0950	64.0 [17.8 to 110.3] p=0.0073

Displays P:F at hour-48 in angiotensin-II vs. placebo when excluding the n=4 patients with exposure to ECMO during the trial period. The Hour-48 columns show the average P:F within the treatment group. The Δ vs. hour-0 columns show the mean difference at Hour-48 vs. baseline within the treatment group, displayed as: Hour-48 – Hour-0. The 95% CI and p-values in these columns reflect the results of a paired T-test. **The baseline-adjusted Δ column reflects the estimate for difference in means by for Angiotensin-II vs. Placebo groups from the linear model adjusted for the hour-0 value (primary analysis)**.
Abbreviations: Δ – difference; SD – standard deviation; ECMO –extracorporeal mechanical oxygenation; 95%CI – 95% confidence interval.

Table S10: Hour-48 P:F Ratio in Angiotensin-II vs. Placebo Groups According to Severity of Hypoxemia at Baseline

	Effect Estimate (Ang-II vs. Placebo)	95% Confidence Interval	p-value
**Stratified Analysis**			
Baseline P:F < 100	+118 mmHg	-219 to +456 mmHg	p=0.43
Baseline P:F 100 to 199	+70 mmHg	-9 to +149 mmHg	p=0.08
Baseline P:F 200 to 299	+66 mmHg	-33 to +166 mmHg	p=0.18
**Continuous Analysis**			
Treatment Effect	+128 mmHg	-50 to +307 mmHg	p=0.16
Interaction Effect *(per 10 mmHg increase in baseline P:F)*	-2 mmHg	-12 to +9 mmHg	p=0.75

Displays the model estimates for the effect of angiotensin-II treatment on hour-48 P:F according to baseline P:F. Effect estimates are reported as the difference for the angiotensin-II group vs. the placebo group. In the stratified analysis, the study cohort was portioned into three subsets based on baseline P:F. In the continuous analysis, the entire cohort was used, and an interaction term was included as a covariate in the model for (baseline P:F)x(treatment group). The interaction effect therefore reflects the change in the effect estimate for angiotensin-II treatment on hour-48 P:F per unit increase in baseline P:F.

Abbreviations: P:F – PaO_2/FiO_2 Ratio; 95%CI – 95% confidence interval

REFERENCES

1. Hughes RA, Heron J, Sterne JAC, Tilling K. Accounting for missing data in statistical analyses: multiple imputation is not always the answer. *Int J Epidemiol*. Aug 01 2019;48(4):1294-1304. doi:10.1093/ije/dyz032

2. Mallinckrodt CH, Clark WS, David SR. Accounting for dropout bias using mixed-effects models. *J Biopharm Stat*. 2001;11(1-2):9-21. doi:10.1081/BIP-100104194
